# Query-Biased Preview over Outsourced and Encrypted Data

**DOI:** 10.1155/2013/860621

**Published:** 2013-09-02

**Authors:** Ningduo Peng, Guangchun Luo, Ke Qin, Aiguo Chen

**Affiliations:** School of Computer Science and Engineering, University of Electronic Science and Technology of China, Chengdu, Sichuan 611731, China

## Abstract

For both convenience and security, more and more users encrypt their sensitive data before outsourcing it to a third party such as cloud storage service. However, searching for the desired documents becomes problematic since it is costly to download and decrypt each possibly needed document to check if it contains the desired content. An informative query-biased preview feature, as applied in modern search engine, could help the users to learn about the content without downloading the entire document. However, when the data are encrypted, securely extracting a keyword-in-context snippet from the data as a preview becomes a challenge. Based on private information retrieval protocol and the core concept of searchable encryption, we propose a single-server and two-round solution to securely obtain a query-biased snippet over the encrypted data from the server. We achieve this novel result by making a document (plaintext) previewable under any cryptosystem and constructing a secure index to support dynamic computation for a best matched snippet when queried by some keywords. For each document, the scheme has *O*(*d*) storage complexity and *O*(log(*d*/*s*) + *s* + *d*/*s*) communication complexity, where *d* is the document size and *s* is the snippet length.

## 1. Introduction

Cloud storage provides an elastic, highly available, easily accessible, and cheap data repository to users who do not want to maintain their own storage or just for convenience, and such a way of storing data becomes more and more popular. In many cases, especially when the users want to store their sensitive data such as business documents, it requires the security guarantees against the cloud provider since an internal staff may access to the data maliciously. Directly encrypting the sensitive documents using traditional encryption techniques such as AES is not an ideal solution since the user will lose the ability to effectively search for the desired documents.

One solution for effectively searching over encrypted data is *searchable encryption* technique. It enables a user to securely outsource his private documents to a third party while maintaining the ability to search the documents by keywords. The scenario is simple: the user submits some encrypted keywords to the server, and then the server performs the search and returns the encrypted documents which contain the queried keywords. However, current searchable encryption techniques either directly return the matched documents or return in the first round some limited information (guided mode) which is prestored in metadata, such as the name and a short static abstract for each matched document. The more documents stored, the more possible matched results will be, and finding the desired documents also becomes a problem. Moreover, the bandwidth cost must be taken into consideration such that returning a large amount of matched documents seemed to be impractical.

Another solution for effectively searching for the desired data is through content preview, which is the main topic of this paper. In modern search engine, if a user searches for a web page by keywords, the search engine will return the name, URI, and a small *query-biased snippet* for each matched page. The snippet explains why such page is matched. Then the user could make a final choice and selectively browse the needed pages without opening all matched links. The same way could be used for searching the desired encrypted documents since the scenario is the same. It could also be combined with searchable encryption to improve the user experience.

However, obtaining a query-biased snippet from an encrypted data is quite challenging. For a general search engine, in order to get a query-biased snippet from a plaintext, it must scan each matched document dynamically, extract the snippets where the keywords occur, then rank the results and finally return the *top-ranking snippet*. While data is encrypted, dynamic scanning becomes quite impossible. Precomputing a snippet file for preview is also impossible because there is no way to know in advance what the queried keywords are, and building all static (keyword, snippet) pairs for each document costs too much storage space even far more than the document itself. Thus, we consider dividing a document to many equal-size encrypted snippets and preconstruct an index to address each snippet. The index stores the information about the keyword frequency in each snippet, which enables the server to dynamically calculate the best snippet for the user when queried by multiple keywords.

There are two major security problems. First, the snippet is the part of a document; therefore the encryption scheme used may affect the snippet retrieval. We use a pad-and-divide scheme to preprocess the document to make it compatible with any cryptosystem such as DES and RSA. Second, the information in the index is private, and no partial information about the document should be leaked to the server. Therefore, we encrypt the index based on the core method of searchable encryption. Since each keyword maps an entry in the index, if queried by some keywords, directly returning the related score information without calculating leaks the information about the number of queried keywords (equals to the number of returned entries) to an eavesdropper, and it also costs multiple communication bandwidth as the number of requested keywords increases. A *homomorphic encryption* scheme could be adopted such that the server could directly operate over the encrypted data and produce a single result, while keeping the ciphertext still secure. However, homomorphic encryption scheme is often costly when dealing with a large amount of data. Observing that all the data are very small, we propose a novel lightweight substitution for homomorphic encryption to construct such secure index.

In this paper, our contributions are the following. (1) To the best of our knowledge, we formalize the problem of securely retrieving query-biased snippet over encrypted data for the first time. We generalize the notion of *secure query-biased preview* (SecQBP) and its security model. (2) We propose a lightweight solution to deal with matrix data with partial homomorphic property, named *matrix additive coding* (Matrix-AC), which could efficiently add two rows of small numbers while keeping the data still encrypted. (3) Based on Matrix-AC and private information retrieval protocol, we construct a *secure additive ranking index* (SecARI) that enables the server to efficiently compute the top-ranking snippet over encrypted data while no partial information about the document is leaked, and then we propose the complete construction to realize SecQBP and prove that it is secure under our security model. (4) We propose a high level solution to combine the preview scheme with searchable encryption technique, which greatly improves the user experience.

The rest of the paper is organized as follows.  Section 2 presents the related work.  Section 3 presents the notations and preliminaries.  Section 4 presents our proposed additive coding scheme. In Section 5 we formally define the preview scheme and its security model and present the construction in detail. We present the application in searchable encryption and analyze the performance in Section 6.  Section 7 concludes this paper.

## 2. Related Work

 We categorize the related work into four topics, and each topic is summarized separately.

### 2.1. Query-Biased Snippet

Query-biased snippet refers to a piece of the content in a document that contains the queried keywords. Query-biased snippet generation schemes are widely used in modern search engine. It is also named *dynamic summary* or *keyword-in-context* (KWIC) snippet generation. The term was used firstly in [1]. The improvements were introduced in [2–6]. However, as far as we know, all query-biased schemes are focused on dynamically retrieving snippets from the plaintext. If the document is encrypted, dynamic scanning becomes impossible. Static preview refers to a snippet summarizing the content in advance, which is always the same regardless of the query. It is generally composed of either a subset of the content or metadata associated with the document. A lightweight static preview scheme over the encrypted data was introduced in [7]. For more details, please refer to [8] for a survey of the recent preview schemes.

### 2.2. Searchable Encryption

Our proposed scheme and security model are based on searchable encryption technique. The basic goal of searchable encryption is to enable a user to privately search over encrypted data by keywords. The first scheme was introduced in [9]. Later on, many index-based symmetric searchable encryption schemes were proposed. The first secure index was introduced in [10], and the security model of adaptive chosen keyword attack (IND-CKA) was also introduced. Reference [11] introduced two constructions to realize symmetric searchable encryption: the first is SSE-1 which is nonadaptive and the second is SSE-2 which is adaptive. A generalization for symmetric searchable encryption was introduced in [12]. Another type of searchable encryption schemes is public-key based. The first scheme was introduced in [13], the improved definition was introduced in [14], and the strongest security model was introduced in [15].

There are many functional extensions for the basic searchable encryption schemes. Reference [16] introduced a scheme supporting conjunctive keyword search. References [17–19] introduced ranked keyword search over encrypted data. References [20–22] introduced fuzzy keyword search over encrypted data. Similar to fuzzy keyword search but different, [23, 24] introduced similarity search over encrypted data.

### 2.3. Homomorphic Encryption

Our proposed additive coding method is based on the core concept of homomorphic encryption. The classical homomorphic encryption schemes are based on group operation such as the unpadded RSA in [25], the variant of ElGamal introduced in [26], Goldwasser and Micali's bit homomorphic encryption scheme introduced in [27, 28], and Paillier's encryption scheme introduced in [29]. Many improvements have been proposed based on these classical series of schemes. The referred schemes are public-key based, and few symmetric homomorphic schemes have been proposed. The series of symmetric homomorphic schemes which is based on one-time pad was introduced in [30]. Some ring-based homomorphic schemes have been proposed recently, which are also referred to as full homomorphic encryption, such as the one in [31] that is based on ideal lattices and the one in [32] that does not require ideal lattices.

### 2.4. Private Information Retrieval

We encapsulate a private information retrieval (PIR) protocol and extend the use of it in our scheme. PIR schemes allow a user to privately retrieve the *i*th bit of an *n*-bit database. The notion was fist introduced in [33] by Chor et al., and the notion of private block retrieval (PBR) was also introduced. Kushilevitz and Ostrovsky introduced a single-server and single-round computational PIR scheme in [34], which achieves communication complexity of $O(\sqrt{n})$ for the basic scheme and could achieve *O*(*n* ^*ϵ*^) with arbitrary small *ϵ* theoretically ($2^{O(\sqrt{\log n\text{loglog}n})}$ is achieved assuming security parameter is polylogarithmic in *n*). In [35], Cashin et al. introduced a single-database PIR scheme with polylogarithmic communication complexity for the first time, about *O*(log⁡^8^ *n*) as suggested. Gentry and Ramzan introduced a PBP scheme with *O*(*k* + *d*) communication cost in [36], where *k* ≥ log⁡*n* is a security parameter that depends on *n*, which is nearly optimal.

## 3. Notations and Preliminaries

### 3.1. Basic Notations

We write *x*←_*U*_ *X* to represent sampling element *x* uniformly random from a set *X* and write *x* ← *𝒜* to represent the output of an algorithm *𝒜*. We write *a*||*b* to refer to the concatenation of two strings *a* and *b*. We write |*A*| to represent its cardinality when *A* is a set and write |*a*| to represent its bit length if *a* is a string. We write ⊕ to represent bitwise exclusive OR (XOR) and “≪*n*” to represent bitwise shift left for *n* bits. We write ⌈*x*⌉ to represent the least integer less than or equal to *x*. We write ${\underline{s}}_{b}$ to represent a bit string that contains either 0 or 1 (e.g., ${\underline{001101}}_{b}$). A function *μ*(*k*) : *ℕ* → ℝ is negligible if for every positive polynomial *p*(·) there exists an inter *N* > 0 such that for all *k* > *N*, |*μ*(*k*)|<1/*p*(*k*). We write poly(*k*) and negl(*k*) to denote polynomial and negligible functions in *k*, respectively.

We write Δ = (*w*_1_,…, *w*_*n*_) to present a dictionary of *n* words in lexicographic order. We assume that all words are of length polynomial in *k*. We write *d* to refer to a document that contains poly(*k*) words. We write $\bar{d}$ to represent the identifier of *d* that uniquely identifies the document, such as a memory location. We write *s* to refer to a snippet (50 characters in general) extracted from the document and write $\bar{s}$ to represent the identifier of *s*, such as the position in the document.

### 3.2. Cryptographic Primitives

A function *f* : {0,1}^*k*^ × {0,1}^*n*^ → {0,1}^*m*^ is pseudorandom if it is computable in polynomial time in *k* and for all polynomial size adversaries *𝒜*, it cannot be distinguished from random functions. If *f* is bijective then it is a pseudorandom permutation. We write the abbreviation PRF for pseudorandom functions and PRP for pseudorandom permutations.

Let ES represent an encryption scheme. Let ES.Gen(1^*k*^) represent the key generation algorithm (*k* is the secure parameter). Let ES.Enc_K_(*d*) represent the encryption algorithm that encrypts data *d* using key *K*, and let ES.Dec_K_(*c*) represent the decryption algorithm that decrypts data *c* to gain the plaintext *d*. In our scheme, a lot of data will be encrypted using the same key; therefore the encryption scheme must be at least CPA (chosen plaintext attack) and CCA (chosen ciphertext attack) secure. For example, ECB (electronic codebook) mode in DES or RSA without OAEP (optimal asymmetric encryption padding) should not be used.

### 3.3. Homomorphism

Let *ℳ* denote the set of the plaintexts, let *𝒞* denote the set of the ciphertexts, let ⊙ denote the operation between the plaintexts and ⊗ the operation between the ciphertexts, and let “←” denote “directly compute” without any intermediate decryption. An encryption scheme is said to be homomorphic if for any given encryption key *k*, the encryption function *E* or the decryption function *D* satisfies

(1) ∀m1,m2∈ℳ, E(m1⊙m2)⟵E(m1)⊗E(m2),

(2) ∀c1,c2∈𝒞, D(c1⊗c2)⟵D(c1)⊙D(c2).

Sometimes, property (2) is also referred to as homomorphic decryption. If the operation is upon a group, we say it is a group homomorphism. If the operation is upon a ring, we say it is a ring homomorphism and is also referred to as full homomorphism. If the operator is addition, we say it is additively homomorphic, and if the operator is multiplication, we say it is multiplicatively homomorphic.

### 3.4. Private Block Retrieval Protocol

Let *B* = (*B*_1_,…, *B*_*n*_) represent a database of *n* blocks; all blocks have equal size *d*. The user wants to privately retrieve the *i*th block from the server; therefore he runs a private block retrieval protocol. At a high level, we define the single database and single round computational PBR as follows.

**Definition (computational PBR protocol)**

A computational PBR protocol scheme is a collection of four polynomial-time algorithms CPBR = (Setup, Query, Response, Decode) such that we have the following.

 
*P*←Setup(*B*) is a probabilistic algorithm that takes as input the database *B* and outputs a parameter set *P*. It is run by the database owner, and *P* is known to all users.
 
*t*←Query(*i*) is a probabilistic algorithm that takes as input a block index *i* and outputs a token *t*. It is run by the user. *t* is sent to the server.
 
*r*←Response(*t*) is a deterministic algorithm that takes as input the requested token *t* and outputs a result *r*. It is run by the server. *r* is sent to the user.
 
*B*_*i*_←Decode(*r*) is a deterministic algorithm that takes as input the response *r* from the server and outputs the requested data block *B*_*i*_. It is run by the user.

In our preview scheme, we adopt the computational PBP scheme as a primitive introduced in [36]. In the setup algorithm, we set the database size as the maximal possible document size (e.g., 10 MB) and reuse prime number set and prime power set in all documents. The communication complexity is *O*(log⁡|*d* | +|*s*|) where |*d*| is the document length and |*s*| is the snippet length.

## 4. Secure Additive Coding

Before introducing the preview scheme, we first introduce a novel coding method called matrix additive coding (Matrix-AC) that enables addition of two rows in a matrix in a homomorphic fashion, which is very fast and suitable for dealing with small numbers (the integer is coded to a specific bit string) and is especially useful for computing statistical table in encrypted form. Since all operated integers are correlative, it is not a homomorphic encryption scheme which could encrypt data independently.

Matrix-AC is used in the preview scheme to construct the secure additive ranking index (SecARI). Becouse a large number of small numbers will be calculated in the preview scheme, using homomorphic encryption schemes is costly. Therefore, we use Matrix-AC scheme as a substitution for homomorphic encryption scheme to achieve optimal performance.

We note that, for all the schemes (including the preview scheme in the next section), we only consider the confidentiality of the data. Mechanism about protecting data integrity is out of the scope of this paper.

### 4.1. Basic Idea

The basic idea of coding small integers *𝕊*_*N*_ = (0,1, 2,…, *N*) with homomorphic property is simple: we consider an integer vector **m** = (*m*_1_,…, *m*_*n*_), where *m*_*i*_ ∈ *𝕊*_*N*_ and ∑_*i*=1_^*n*^ *m*_*i*_ ≤ *N*. We define a “vernier” that has *N* bits, and each integer *m*_*i*_ is mapped to such vernier for *m*_*i*_ bits in different position. A global cursor *g* is autoincreased to process the mapping. To code a message, a random string as a one-time-pad key is used and XORed with the mapped data. The decoding is simple: just operate ⊕ with the key and count the number of bit-1 to make reverse mapping.

**Figure 1:**
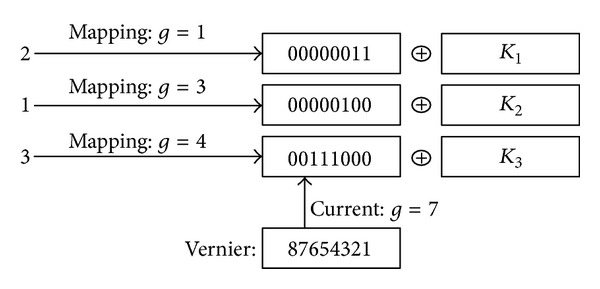
Example of vernier mapping and the basic coding procedure.

For example (as shown in Figure 1), consider a vernier that has 8 bits, and we map three integers (2,1, 3) as a vector to three pairs ($2,{\underline{00000011}}_{b}$), ($1,{\underline{00000100}}_{b}$), and ($3,{\underline{00111000}}_{b}$). It is easy to see that the homomorphic property holds as 2 + 3 = 5, and ${\underline{00000011}}_{b}⊕{\underline{00111000}}_{b}={\underline{00111011}}_{b}$ which has exactly 5 bit-1. Thus, let the vector be (*m*_1_ = 2, *m*_2_ = 1, *m*_3_ = 3), and let the keys be (*K*_1_, *K*_2_, *K*_3_) that each key is a random string; then the ciphertext vector will be $(c_{1}={\underline{00000011}}_{b}⊕K_{1}$, $c_{2}={\underline{00000100}}_{b}⊕K_{1}$, $c_{3}={\underline{00111000}}_{b}⊕K_{1})$. To perform addition for any two plaintexts *m* = *m*_1_ + *m*_2_, the server could directly compute the corresponding ciphertexts *c* = *c*_1_ ⊕ *c*_2_ and the decryption key becomes *K* = *K*_1_ ⊕ *K*_2_. Using the new key, it is easy to decrypt the ciphertext and count the number of bit-1 to restore the result.

The problem of the basic scheme is that the vernier may be used up. That is why we set the restriction that a vernier is just used in a single vector. Another drawback is that, as the max *N* increases, the length of the vernier also increases in linear *O*(*N*). Thus, the targeted data must be small enough to save storage space. A good application is not for dealing with few such integers but for computing a large number of small data in parallel.

### 4.2. Coding a Matrix

We extend the basic idea to code the data matrix. Let *A*_*m*×*n*_ represent a matrix with *m* rows and *n* columns, let *a*_*ij*_ represent the element in row *i* and column *j*, and *a*_*i*_ represent the *i*th row. Matrix-AC scheme is described in Algorithm 1. Note that there are *n* cursors that control the mapping for each column.

Let us check the homomorphism for decoding: let *D* represent the decryption algorithm, for arbitrary two ciphertext rows *c*_1_ and *c*_2_, *D*(*c*_1_ ⊕ *c*_2_) = *D*(*c*_1_) + *D*(*c*_2_), where the decryption key for *c*_1_ ⊕ *c*_2_ is *K*_1_ ⊕ *K*_2_.

There is a problem if the scheme is directly used in the application. In the real world, there is no way to directly represent, for example, data of 5 bits (there is an extended “bitset” class in C++, but it treats the bits as a set, and all operations are performed over set, and it is very slow). In computer, the data is represented by “byte” that a valid number is stored in such a byte. Thus, 5-bit data is stored in one byte (8 bits) as a “character,” 12-bit data is stored in two bytes (16 bits) as a “short integer,” and a 20-bit data is stored in four bytes (32 bits) as an “integer.” Thus, the XOR operation is performed over byte, and the data should be extended to such standard length. However, since all data in Matrix-AC are in fact a bit string, sometimes the data in the same row could be “chained” together. For example, suppose *N* = 5 and there are 6 data in a row; the row could be chained to a 30-bit string and stored in a 32-bit integer. Another problem is that the “bit-counting” algorithm is realized indirectly by “mod 2” operation or setting *N* masks to see if the masked bit is 1. Therefore, the performance would be improved if some dedicated hardware directly dealing with bits is used.

### 4.3. Proof of Security

Intuitively, the scheme is secure if any two matrices (the numbers of elements are the same) prepared by the adversary are indistinguishable, which also implies that any two elements from the same matrix are indistinguishable. We define the security of Matrix-AC as follows.

**Lemma**

If *K*_1_,…, *K*_*n*_ are random strings, then Matrix-AC is CPA secure.

**Proof**

We briefly prove the scheme since the mechanism is simple. We describe a PPT simulator *𝒮* for all PPT adversaries *𝒜*. *𝒮* generates matrix **X**_*m*×n_* with *m* random strings of length *n* · *N*. For any row *x*_*i*_ in the original matrix **X**_*m*×*n*_, no matter how it maps, the last computation is a string XORed with a one-time-pad random string *K*_*i*_; thus the result is indistinguishable from random. For any two rows *x*_*i*_ and *x*_*j*_ from the same matrix, each row is XORed with different random strings such that the results are indistinguishable from each other. For any two rows *x*_*i*_ and *x*_i_* from two matrices, as discussed previously, *x*_*i*_ is indistinguishable from the random string *x*_i_*.

## 5. Secure Query Biased Preview Scheme

The preview scheme contains two steps: (1) storage at which the data owner prepares the previewable document and a searchable index; (2) retrieval at which the user privately retrieves the snippet from the server.

The basic idea of constructing a query-biased previewable document is as follows: divide the document into *n* snippets with equal size, extract keywords from each piece to form a keyword set which records the snippet information as (keyword, frequency) pairs, and build an index to address the snippets according to the distinct keywords. The index *R* is a *m* × *n* two-dimensional matrix of the form *R*(keyword, snippet index), and the value is the keyword frequency in the corresponding snippet. An example is shown in Table 1. The keyword is represented by *w*_*i*_, and the snippet index is represented by *s*_*i*_.

The main process of retrieving the best snippet by multi keywords follows the following steps. The user submits multikeywords to the server. The server retrieves the multirows in the index according to the submitted keywords and adds the rows together. The result is a single entry that contains the information about the best matched snippet. The user decrypts the entry, selects the snippet identifier (index number) with the highest score (for simplicity, the score equals the frequency), and privately retrieves the snippet from the server by running a PBR protocol. In order for the server to perform the “addition” operation over the encrypted data, a homomorphic encryption scheme could be used to encrypt the index. We adopt Matrix-AC as the encryption scheme instead of a standard homomorphic encryption scheme as discussed previously.

Now we begin to introduce the definition and the security model of the preview scheme. Note that we assume the server is honest but curious. Additional methods could be added to make those solutions robust against malicious attack; however, we restrict our discussion on honest-but-curious fashion. We also note that all documents are treated as text files the same way as search engine does. For example, if a document is a web page, the style tags will be pruned.

### 5.1. Scheme Definition

The secure-query biased preview (SecQBP) scheme contains two parties: a user *U* and a remote server *S*. *U* encrypts his private document *d* to *D*, generates a secure additive ranking index (SecARI) *H*, and then outsources them to *S*. *S* stores the document, performs the computation for the scores when queried by multiple keywords, and returns the result to *U*. *U* then selects the best snippet indexed by *i* and privately retrieves it from *S*.

Without loss of generality, we consider the construction for a single document. The scheme could be extended to a document collection with ease. Now we define the SecQBP scheme as follows.

**Definition (secure query-biased preview scheme)**

SecQBP scheme is a collection of six polynomial-time algorithms SecQBP = (Gen, Setup, Query, ComputeScore, DecScore, DecSnip) as follows.

 
*K*←Gen(1^*k*^) is a probabilistic algorithm that takes as input a security parameter *k* and outputs the secret key collection *K*. It is run by the user, and the keys are kept secret.
 (*D*, *H*)←Setup_K_(*d*) is a probabilistic algorithm that takes as input a document *d* and outputs a encrypted document *D* (using any cryptosystem) and an index *H*. It is run by the user, and *D*, *H* are outsourced to the server.
 
*q*←Query_K_(**w**) is a deterministic algorithm that takes as input the queried multiple keywords **w** = (*w*_1_,…, *w*_*n*_) and outputs a secret query token *q*. It is run by the user, and *q* is sent to the server.
 
*r*←ComputeScore(*q*, *H*) is a deterministic algorithm that takes as input the secret query *q* and the index *H* and outputs the result *r* that contains the final score information about each snippet. It is run by the server.
 
*i*←DecScore_K_ $\left(w,\overset{-}{d},r\right)$ is a deterministic algorithm that takes as input the queried keywords **w**, the document identifier $\bar{d}$, and the query result *r* and outputs the snippet index number *i*. It is run by the user.
 
*s*_*i*_←DecSnip_K_(*D*_*i*_) is a deterministic algorithm that takes as input the ciphertext *D*_*i*_ and outputs the recovered plaintext snippet *s*_*i*_. It is run by the user. Note that, if the user retrieves the entire encrypted document, he could decrypt the document by decrypting each snippet.

### 5.2. Security Model

Informally speaking, SecQBP must guarantee that, first, given the encrypted document *c* and the index *H*, the adversary cannot learn any partial information about the document; second, given a sequence of queries *q* = (*q*_1_,…, *q*_*n*_), the adversary cannot learn any partial information about the queried keywords and the matched snippet (including the index number and the content). We now present the security definition for adaptive adversaries.

**Definition (semantic security against adaptive chosen keyword attack, CKA2-security)**

Let ∑ = (SecQBP algorithm + SecQBP protocol) be the preview scheme. Let *k* ∈ *ℕ* be the security parameter. one considers the following probabilistic experiments, where *𝒜* is an adversary and *𝒮* is a simulator.

 Real_Σ,*𝒜*_(*k*): the challenger runs Gen(1^*k*^) to generate the key *K*. *𝒜* generates a document *d* and receives (*D*, *H*)←Setup_K_(*d*) from the challenger. *𝒜* makes a polynomial number of adaptive queries **w**_1_,…, **w**_*n*_ (each set **w**_*i*_ contains multiple keywords in *d*), and for each queried keyword set **w**_*i*_, *𝒜* receives a query token *q*_*i*_←Query_K_(**w**_*i*_) from the challenger. Finally, *𝒜* returns a bit *b* that is output by the experiment.
 Sim_Σ,*𝒜*,*𝒮*_(*k*): *𝒜* generates a document *d*. Given only the size |*d*|, *𝒮* generates and sends (D*, H*) to *𝒜*. *𝒜* makes a polynomial number of adaptive queries **w**_1_,…, **w**_*n*_ (each set **w**_*i*_ contains multiple keywords in *d*), and for each queried keyword set **w**_*i*_, *𝒜* receives a query token *q*_i_* from *𝒮*. Finally, *𝒜* returns a bit *b* that is output by the experiment.

We say that SecQBP is semantic secure against adaptive chosen keyword attack if, for all PPT adversaries *𝒜*, there exists a PPT simulator *𝒮* such that

(3) |Pr[Real∑,𝒜(k)=1]−Pr[Sim∑,𝒜,𝒮(k)=1]|≤negl(k),

where the probabilities are over the coins of Gen and Setup (related to the underlying cryptosystem).

Note that, with *q*_*i*_ or *q*_i_*, *𝒜* could run ComputeScore(*q*, *H*) to get the result *r*, and any internal state is also captured by *𝒜*. *𝒜* could also send query according to the previous result.

### 5.3. Concrete Construction

Now we describe the concrete construction for SecQBP. We describe the constructions for some core components, and then represent the complete construction.

#### 5.3.1. Encrypting a Document

We consider the problem of extracting keywords from a document. In general, a keyword is followed by a separator. Thus, in a general snippet of 50 characters, no more that 25 keywords are contained. Another problem is that not all words are keywords, and such words do not need indexing, for instance, the words “a,” “the,” and “and.” This kind of words can be found in most of the sentences such that it is useless as a key to index a file. They are called stop-word and firstly researched in [37]. The most classical stop word list used abroad is a list of 425 words suggested in [38].

There is a problem that the last word in a snippet may be cut off. In other words, the last word of a snippet may be not short enough to fit the space, and it cannot be split into two words because neither of them is a valid keyword. In a general search engine, such overflowed word is omitted. However, in the scenario of precomputing snippets, if the word is omitted, a keyword may be lost. It means that, when querying the omitted keyword, there will be no matched snippet returned, where actually there is a match for the document. Thus, we add the full word to both the keyword sets of the snippets which contain part of the keyword.

The basic idea for encrypting a document is dividing the document with equal size; therefore, a padding scheme is needed when the last piece of the document is not long enough. We modify the CBC plaintext padding scheme introduced in [39] to meet our goal. Let |*s*| represent the length of the snippet; the snippet is treated as a sequence of bytes. If the last snippet is *a* bytes, then pad the snippet with |*s* | −*a* bytes with value |*s* | −*a*. After decryption, the padding will be deleted to recover the original plaintext. For instance, suppose |*s* | = 50; if the final snippet has 15 plaintext bytes, then pad the snippet as

(4) byte1||byte2||⋯||byte15||35||35||⋯||35,

where there are 35 bytes that have the number 35. If the snippet is divisible by |*s*|, here is 50, then add a new snippet with all bytes being 50:

(5) 50||50||⋯||50.

Let *d* represent a document, and *D* is the encrypted form of *d*. We introduce the scheme for encrypting a document, shown in Algorithm 2. In the algorithm, “a valid keyword” means the token is not a separator, not a stop word, and not a random-looking string. A word dictionary could be used to check its validity.

#### 5.3.2. Constructing the Secure Index

The secure additive ranking index (SecARI) is the encryption form of the snippet index, as shown in Table 1 (PAD denotes the padding with a random string), and each row is an encrypted entry. For security reason, the number of entries of SecARI must be padded to a certain amount which is independent of the actual number of keywords in the content, or it will leak the information about the number of distinct keywords in the document (it equals the number of rows). An example of a SecARI is shown in Table 2. In the table, *π* is a pseudorandom permutation which randomizes the order of the keywords, and the value *c*_*ij*_ is the encrypted score.

Let us consider the secure amount of the entries. If the document *d* is small, let a keyword occupy only one byte; then the maximum possible number of keywords is |*d* | /2 (as discussed, a valid keyword is at least 2 bytes); thus, the number of entries must be set to |*d* | /2 (the fractional part is ignored). If the document *d* is large, the maximum possible number of keywords equals the total number of words in the dictionary. Reference [40] made a detailed word statistical analysis based on 450 million words on Corpus of Contemporary American English (1990–2012). The statistics show that the total words used are about 60000. We set the dictionary used as Δ and define the maximum keyword amount as |Δ | = 60000. Thus, we define the number of entries as follows.

**Definition (number of entries)**

To guarantee security, the number of entries *N*_ent_ for a SecARI is

(6) Nent={|d|2,|d|2<|Δ|,|Δ|,|d|2≥|Δ|.

SecARI is in fact a sparse look-up table, and we use indirect addressing method to manage it. Indirect addressing method is also called *FKS dictionary* introduced in [41], which is also adopted in symmetric searchable encryption scheme in [11]. It manages sparse table of the form (address, value). The address is a *virtual address* that could locate the value field. Given the address, the algorithm will return the associated value in constant look-up time and return *Ø* otherwise.

In addition, we make use of a pseudorandom permutation *π* to index an entry and a pseudorandom function to generate the one-time-pad keys for Matrix-AC:

(7) π:{0,1}k×{0,1}|w|→{0,1}|w|,f:{0,1}k×{0,1}|w|+|d¯|→{0,1}N·n,

where |*w*| is the keyword length and $\left|\bar{d}\right|$ is the length of the document identifier. *N* is the upper bound discussed in Matrix-AC and *n* is the number of snippets that is calculated from the document size. The submitted keyword is encrypted by *π* such that the server cannot figure out what the keyword the user queries.

Let *H* be a {0,1}^|*w*|^ × {0,1}^*N*·*n*^ × *N*_ent_ data matrix managed by indirect addressing technique as discussed previously. Now we describe SecARI in Algorithm 3.

#### 5.3.3. The Complete Scheme

In order to hide the information about the number of queried keywords, a SecARI is not enough. When the user submits the queried multiple keywords, each query should be of the same length so that an eavesdropper cannot learn the information about the number of keywords in a query. Let the maximum number of keywords allowed in a single query be *W*_max⁡_; the remaining space must be padded. The user and the server should initiate a secure channel such as SSL to transport such message, or the padding may be discovered by an eavesdropper. Since the size of a keyword is small, the bandwidth waste of the padding is rather negligible.

We also determine the upper bound *N* for Matrix-AC. As discussed, a general snippet contains at most 25 keywords; thus we set *N* = 32 (stored as a standard integer).

Let *f* be the pseudorandom function, and *π* is the pseudorandom permutation as described previously. Now we describe the complete scheme in Algorithm 4, and describe the storage and retrieval protocol in Protocol 1. The retrieval protocol describes the retrieval of a query-biased snippet from document *d* by submitting a multikeyword query **w** = (*w*_1_,…, *w*_*n*_).

**protocol 1:**
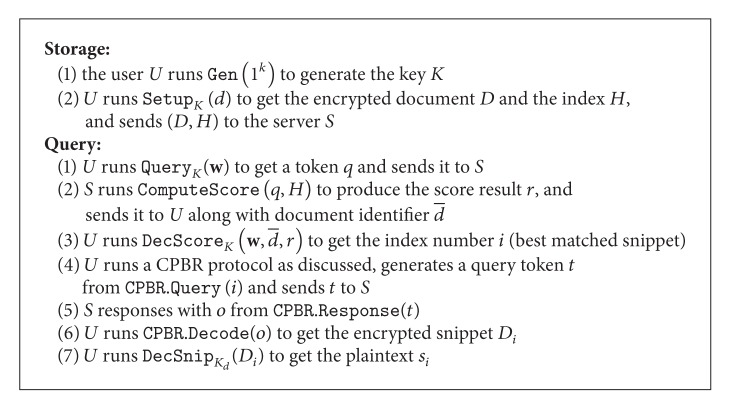
SecQBP protocol.

Note that it is a scenario for a single document. The protocol also works for a document collection. Thus, the user could retrieve multiple snippets for multiple documents in the same round.

### 5.4. Proof of Security

The server stores the SecARI, performs homomorphic computation for a query, and returns to the user the score information as a single entry. We prove the security by introducing a theorem as follows.

**Theorem**

If *f* is a pseudorandom function, if *π* is a pseudorandom permutation, and if ES is CPA and CCA secure, then SecQBP is CKA2 secure.

**Proof**

We describe a polynomial-size simulator *𝒮*, for all polynomial-size adversaries *𝒜*, Real_∑,*𝒜*_(*k*) and Sim_∑,*𝒜*,*𝒮*_(*k*) are indistinguishable. Consider the simulator that given the size of the document |*d*|, *𝒮* generates the data as follows.

1. (Simulating H*) *𝒮* computes *m* = *N*_ent_, *n* = ⌈|*d* | /|*s*|⌉. For 1 ≤ *i* ≤ *m*, *𝒮* generates a string *a*_i_*||*c*_i_* such that each *a*_i_* is a distinct string of length |*w*| chosen uniformly at random, and each *c*_i_* is a string of length *N* · *n* bits chosen uniformly at random. All strings form H*.
2. (Simulating *q*_i_*) *𝒮* prepares a query list *L* that stores the query history. The value in *L* is of the form (*w*, a*). When queried by a keyword set **w**_*i*_, for each keyword *w*_*k*_ in **w**_*i*_, *𝒮* first scans *L* to see if there is a match. If not, *𝒮* randomly chooses a distinct *a*_k_* which is not in *L* and stores the pair (*w*_*k*_, *a*_k_*) into *L*. *𝒮* gets (*a*_1_*,…, *a*_|**w**_*i*_|_*) according to **w**_*i*_ and sets *q*_i_* = (*a*_1_*,…, *a*_|**w**_*i*_|_*).
3. (Simulating *D*_i_*) *𝒮* sets *D*_i_* to a |*D*_*i*_|-bit string chosen uniformly at random. Note that |*D*_*i*_| is a global parameter known by the user and the server.

We claim that no polynomial-size distinguisher *𝒟* could distinguish the following pairs.

1. (*H* and H*) recall that *H* consists of *N*_ent_ values. Each value consists of either a string of the form (*π*_*K*_*p*__(*w*_*i*_)||*c*_*i*_) or a random string. In any case, with all but negligible probability, the PRP key *K*_*p*_ is not included; therefore the pseudorandomness of *π* guarantees that *π*_*K*_*p*__(*w*_*i*_) is indistinguishable from random. The PRF key *K*_*m*_ is also not included; therefore the pseudorandomness of *f* guarantees that the derived key *k*_*i*_ for each data row is indistinguishable from random, and then the underlying Matrix-AC is CPA-secure, which means that *c*_*i*_ is indistinguishable from random. H* contains *N*_ent_ random values. Therefore, as discussed, *H* and H* are indistinguishable.
2. (*q*_*i*_ and *q*_i_*) recall that *q*_*i*_ is the evaluation of the PRP *π*. In any case, with all but negligible probability, the PRP key *K*_*p*_ is not included; therefore the pseudorandomness of *π* guarantees that all *π*_*K*_*p*__(*w*_*i*_) in *q*_*i*_ are indistinguishable from random, and *q*_i_* is a random string of the same length of *q*_*i*_.
3. (*D*_*i*_ and *D*_i_*) recall that *D*_*i*_ is encrypted by a CPA and CCA secure encryption scheme. Since the encryption key *K*_*d*_ is not known by the adversary, the security of the encryption scheme guarantees that *D*_*i*_ and *D*_i_* are indistinguishable.

## 6. Comparison, Application, and Performance Analysis

First, we compare the functionalities and performance of our work with previous works. Then, as a significant example, we discuss how to combine the preview scheme with symmetric searchable encryption to improve the user experience. We also discuss the performance of the preview scheme in the concrete application example.

### 6.1. Scheme Comparison

Let *s* denote the snippet length and *d* the document size; the comparisons of our work with other representative works are shown in Table 3.

The query-biased preview mode is widely used in general search engine, as introduced in [5]. In the scheme, the search engine dynamically scans the document line by line to find the top-ranking snippet. Therefore, the computation complexity is *O*(*d*). In [7], Mithal and Tayebi proposed a static preview scheme over encrypted data based on content mask technique. In the scheme, some segments of the plaintext are extracted in advance and are masked with noise in such a way that the so called “masked preview content” could be sent to the user as a preview when queried. The static scheme is fast and informative but does not explain why a document is matched by a query. Note that our scheme costs one extra round of communication since the score results have to be returned to the user in the first round.

### 6.2. Symmetric Searchable Encryption Extension

We review the generalized definition of symmetric searchable encryption (SSE) introduced in [12]. We assume that the searchable encryption scheme is in guided mode. In other words, the server will first return to the user the identifiers of the matched documents, and the user makes a final choice to select some document identifiers and sends them to the server to retrieve the selected ones.

**Definition (extended symmetric searchable encryption)**

In guided mode, a symmetric searchable encryption scheme is a collection of six polynomial-time algorithms SSE = (Gen, Enc, Token, Search, Retrieve, Dec) such that we have the following.

 
*K* ← Gen(1^*k*^) is a probabilistic algorithm that takes as input a security parameter *k* and outputs a secret key *K*. It is run by the user, and the output key is kept secret by the user.
 (*γ*, *C*) ← Enc_*K*_(*D*) is an algorithm that takes as input a secret key *K* and a document collection *D* = (*D*_1_,…, *D*_*n*_) and outputs a searchable structure *γ* and a sequence of encrypted documents *C* = (*C*_1_,…, *C*_*n*_). It enables a user to query some keywords, and the server returns the matched documents. For instance, in an index-based searchable symmetric encryption scheme, *γ* is the secure index. It is run by the user, and (*γ*, *C*) is sent to the storage server.
 
*t* ← Token_*K*_(**w**) is a deterministic (possibly probabilistic) algorithm that takes as input a secret *K* and a set of some keywords **w** = (*w*_1_,…, *w*_*n*_) and outputs a search token *t* (also named trapdoor or capacity). It is run by the user.
 
*I* ← Search(*γ*, *t*) (guided mode) is a deterministic algorithm that takes as input the query token *t* and the searchable structure *γ* and outputs the matched document identifiers *I* = (*I*_1_,…, *I*_*m*_). It is run by the server, and the result *I* is sent to the user. Note that, if not in guided mode, this algorithm returns the matched documents directly. It is run by the server.
 
*C*′ ← Retrieve(*C*, *I*′) is a deterministic algorithm that takes as input the encrypted documents and the selected document identifiers *I*′⊆*I* and outputs the selected documents corresponding to the identifiers. It is run by the server.
 
*D*_*i*_ ← Dec_*K*_(*C*_*i*_) is a deterministic algorithm that takes as input a secret key *K* and the returned encrypted document *C*_*i*_ and outputs the recovered plaintext *D*_*i*_. It is run by the user.

The preview scheme is applied in SSE as follows. The user runs SSE.Gen, SecQBP.Gen, SSE.Enc, and SecQBP.Setup, respectively. The server stores the outsourced structure generated by SSE and the encrypted documents generated by SecQBP scheme. To search for some documents, the user runs SSE.Token and SecQBP.Query, respectively, and sends them to the server. The server produces the identifiers of the matched documents, runs SecQBP.ComputeScore for the corresponding documents one by one, and returns the document identifiers and the score results together. The user decodes the score, retrieves the preview snippets from the server, then makes the choice, and sends the selected document identifiers to the server to retrieve the interested documents.

### 6.3. Performance Analysis

We adopt SSE-2 introduced in [11] as an instance of a SSE scheme.  Table 4 shows the time complexity and storage complexity for single SSE-2 scheme and SSE-2 plus SecQBP in detail.

Let *C* represent the encrypted document collection, so the total size is |*C*| bytes. Other than the returned encrypted documents, the extrastorage cost for SSE-2 is |*C* | /8 bytes; thus the storage cost is *O*(*n*). The extrastorage cost for SecQBP is *H* for each document. By definition, the storage cost is *O*(*n*). For SSE, the server searches the matched documents and decrypts the identifier list. For SecQBP, the server searches the indices for all matched documents, returns score results for all matched documents, and finally returns the snippets. They are both in time complexity of *O*(1). The number of rounds for SSE is two (guided mode). First, the server returns the identifiers of the matched documents and next returns the selected documents. SecQBP adds extra round for retrieving snippets from the snippet server. Moreover, for each matched document, the size of the messages for SEE is *O*(1). SecQBP is *O*(log⁡(*d*/*s*) + *s* + *d*/*s*), where *d* is the document length, and *s* is the snippet length, since the user will receive a score result of size *d*/*s* and a snippet of size *s*.

The detailed performance of SSE is analyzed in [42]; therefore we just analyze the performance of the SecQBP part. The content of a document *d* is varied in the real world. By observation in [40], the number of keywords in a document increases along with the document size which satisfies log model, and the worst case satisfies linear model (each word in the document is keyword, such as a dictionary). However, the design for security in our scheme guarantees that the encrypted indices generated from any document are indistinguishable. Therefore, the computation for the server is independent from the models (i.e., the computations for all documents are the same). To simulate the reality, we design the data generator that simulates documents using log model.

In order to demonstrate the optimization for the server, we compare our suggested Matrix-AC scheme with the simplest and, as far as we know, the fastest symmetric homomorphic encryption scheme [30] denoted by SHE and a well-known homomorphic cryptosystem [29] denoted by Paillier cryptosystem. We consider that 100 users submit queries simultaneously. Each query contains 5 keywords, and the score computation is over 100 matched documents (SSE generates the identifiers of the matched documents). The size of each document increases from 50 KB to 1 MB (the sizes for all stored documents are the same), and the computation cost is described by millisecond.

**Figure 2:**
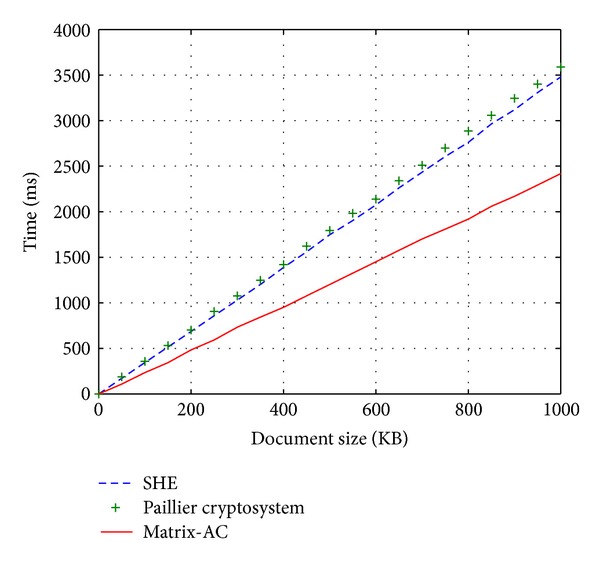
Time cost for computing scores (single server, 100 users).

 The algorithms are coded in C++ programming language and the server is a Pentium Dual-Core E5300 PC with 2.6 GHz CPU. The result is shown in Figure 2. It demonstrates that:the following. (1) The scheme is secure. The figure shows a linear computation cost, which means the computation is independent of the document content. In other words, the server does not see any differences for all documents while performing the search. (2) In cloud environment, computation for 100 users simultaneously on a single server becomes a burden as the size of the document increases. In other words, the number of servers run as services is determined by the size of the stored documents and the accepted queries. (3) The performance is improved as we adopt Matrix-AC to substitute the homomorphic encryption schemes. From the data, Matrix-AC is about 30% faster than using SHE or Paillier cryptosystem. We assume that the user does not modify the document frequently, and the main operation is just searching for some documents. Therefore, the performance improvement is significant since it could save about 30% virtual machines in the cloud.

## 7. Conclusions

In this paper, we propose a generalized method of securely retrieving query-biased snippet over outsourced and encrypted data, which allows the users to take a sneak preview over their encrypted data. The preview scheme has strong security and privacy guarantees with relatively low overhead, and it greatly improves the user experience.

## Figures and Tables

**Algorithm 1:**
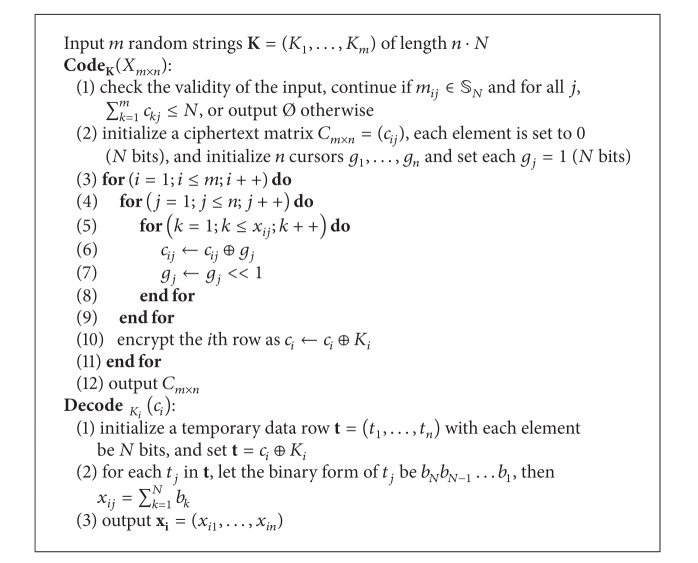
Secure additive coding for matrix.

**Algorithm 2:**
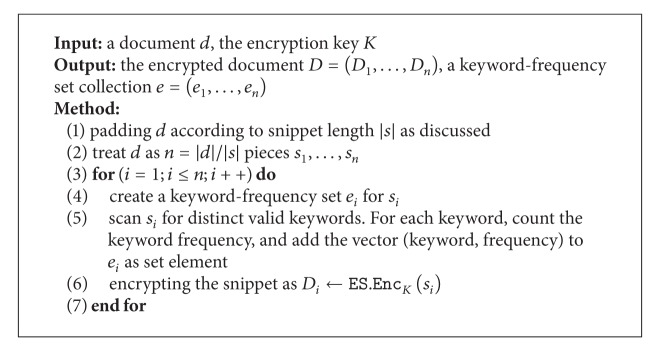
Encrypting a document: ${ED}_{K}(d)$.

**Algorithm 3:**
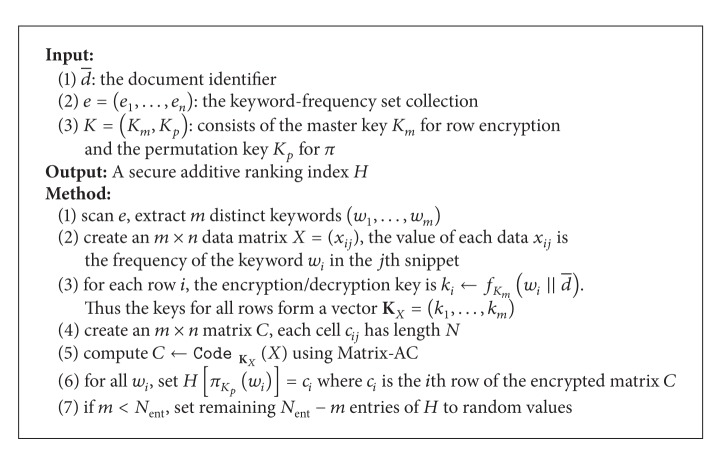
Constructing SecARI: ${Index}_{K}\left(\bar{d},e\right)$.

**Algorithm 4:**
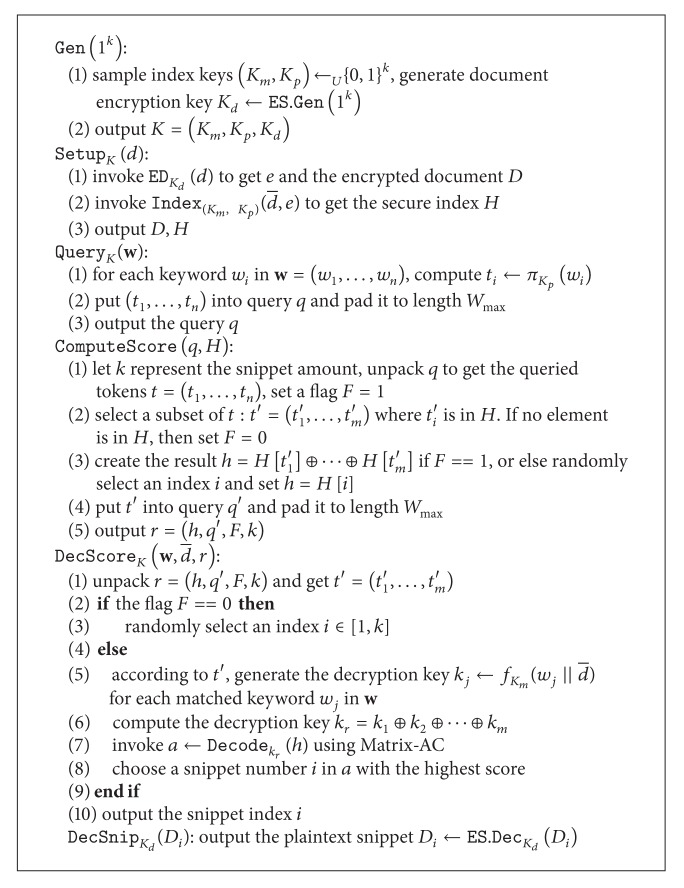
SecQBP algorithm.

**Table 1:** Example of a snippet index.

Keyword	*s*_1_	*s*_2_	*⋯*	*s*_*n*_
*w*_1_	2	1	*⋯*	2
*w*_2_	1	2	*⋯*	1
*⋯*	*⋯*	…	*⋯*	*⋯*
*w*_*i*_	2	5	*⋯*	3
*⋯*	*⋯*	…	*⋯*	*⋯*
*w*_*m*_	3	2	*⋯*	2

**Table 2:** Example of a SecARI.

Index	*s*_1_	*s*_2_	*⋯*	*s*_*n*_
*π*_*K*_(*w*_2_)	*c*_21_	*c*_22_	*⋯*	*c*_2*n*_
*⋯*	*⋯*	*⋯*	*⋯*	*⋯*
PAD	PAD	PAD	PAD	PAD
*π*_*K*_(*w*_1_)	*c*_11_	*c*_12_	*⋯*	*c*_1*n*_
*⋯*	*⋯*	*⋯*	*⋯*	*⋯*
*π*_*K*_(*w*_*m*_)	*c*_*m*1_	*c*_*m*2_	*⋯*	*c*_*mn*_
PAD	PAD	PAD	PAD	PAD
*π*_*K*_(*w*_3_)	*c*_31_	*c*_32_	*⋯*	*c*_3*n*_
*⋯*	*⋯*	*⋯*	*⋯*	*⋯*

**Table 3:** Comparisons of preview schemes.

	Data type	Preview mode	Round	Communication	Storage	Computation
General search engine [5]	Plaintext	Query biased	1	*O*(*s*)	*d*	*O*(*d*)
Content mask [7]	Plaintext or ciphertext	Static	1	*O*(*s*)	*O*(*d*)	*O*(1)
Our scheme	Ciphertext	Query biased	2	*O*(log⁡⁡(*d*/*s*) + *s* + *d*/*s*)	*O*(*d*)	*O*(1)

**Table 4:** Properties of SSE-2 + SecQBP.

Properties	SSE-2	SSE-2 + SecQBP
Adaptive adversaries	Y	Y
Number of servers	1	1
Server storage	*O*(*n*)	*O*(*n*)
Server computation	*O*(1)	*O*(1)
Number of rounds	2	3
Extracommunication	*O*(1)	*O*(log⁡⁡(*d*/*s*) + *s* + *d*/*s*)

## References

[1] Tombros A, Sanderson M Advantages of query biased summaries in information retrieval.

[2] Christopher D, Manning PRHS (2008). *Introduction To Information Retrieval*.

[3] Goldstein J, Kantrowitz M, Mittal V, Carbonell J Summarizing text documents: sentence selection and evaluation metrics.

[4] Sakai T, Sparck-Jones K Generic summaries for indexing in information retrieval.

[5] Turpin A, Tsegay Y, Hawking D, Williams HE Fast generation of result snippets in web search.

[6] White RW, Ruthven I, Jose JM Finding relevant documents using top ranking sentences: an evaluation of two alternative schemes.

[7] Mithal AK, Tayebi A (2009). *Method and System For Facilitating Search, Selection, Preview, Purchase Evaluation, Offering For Sale, Distribution, and/or Sale of Digital Content and Enhancing the Security Thereof*.

[8] Nenkova A, McKeown K (2012). A survey of text summarization techniques. *Mining Text Data*.

[9] Song DX, Wagner D, Perrig A Practical techniques for searches on encrypted data.

[10] Goh EJ (2003). Secure indexes. http://eprint.iacr.org/2003/216.

[11] Curtmola R, Garay J, Kamara S, Ostrovsky R Searchable symmetric encryption: Improved definitions and efficient constructions.

[12] Chase M, Kamara S Structured encryption and controlled disclosure.

[13] Boneh D, Crescenzo GD, Ostrovsky R, Persiano G Public key encryption with keyword search.

[14] Abdalla M, Bellare M, Catalano D Searchable encryption revisited: consistency properties, relation to
anonymous ibe, and extensions.

[15] Boneh D, Kushilevitz E, Ostrovsky R, Skeith WE Public key encryption that allows pir queries.

[16] Golle P, Staddon J, Waters B Secure conjunctive keyword search over encrypted data.

[17] Cao N, Wang C, Li M, Ren K, Lou W Privacy-preserving multi-keyword ranked search over encrypted cloud data.

[18] Wang C, Cao N, Li J, Ren K, Lou W Secure ranked keyword search over encrypted cloud data.

[19] Swaminathan A, Mao Y, Su G-M Confidentiality-preserving rank-ordered search.

[20] Bosch C, Brinkman R, Hartel P, Jonker W Conjunctive wildcard search over encrypted data.

[21] Bringer J, Chabanne H Embedding edit distance to allow private keyword search in cloud computing.

[22] Li J, Wang Q, Wang C, Cao N, Ren K, Lou W Fuzzy keyword search over encrypted data in cloud computing.

[23] Cong W, Kui R, Shucheng Y, Urs KMR Achieving usable and privacy-assured similarity search over outsourced cloud data.

[24] Kuzu M, Islam MS, Kantarcioglu M Efficient similarity search over encrypted data.

[25] Rivest RL, Shamir A, Adleman L (1983). A method for obtaining digital signatures and public-key cryptosystems. *Communications of the ACM*.

[26] Singh AK, Chandran P A secure and efficient multi-authority proactive election scheme.

[27] Goldwasser S, Micali S (1982). Probabilistic encryption & how to play mental poker keeping secret all partial information. *Annual ACM Symposium on Theory of Computing*.

[28] Goldwasser S, Micali S (1984). Probabilistic encryption. *Journal of Computer and System Sciences*.

[29] Paillier P Public-key cryptosystems based on composite degree residuosity classes.

[30] Castelluccia C, Mykletun E, Tsudik G Efficient aggregation of encrypted data in wireless sensor networks.

[31] Gentry C Fully homomorphic encryption using ideal lattices.

[32] Van Dijk M, Gentry C, Halevi S, Vaikuntanathan V (2010). Fully homomorphic encryption over the integers. *Lecture Notes in Computer Science*.

[33] Chor B, Goldreich O, Kushilevitz E, Sudan M (1998). Private information retrieval. *Journal of the ACM*.

[34] Kushilevitz E, Ostrovsky R Replication is not needed: single database, computationally-private information retrieval.

[35] Cashin C, Micali S, Stadler M Computationally private information retrieval with polylogarithmic communication.

[36] Gentry C, Ramzan Z Single-database private information retrieval with constant communication rate.

[37] Luhn HP (1969). A statistical approach to mechanized encoding and
searching of literary information. *Pioneer of Information Science, Selected Works*.

[38] Fox C (1992). Lexical analysis and stop-lists. *Information Retrieval: Data Structures and Algorithms*.

[39] Rivest RL, Baldwin RW (1996). The rc5, rc5-cbc, rc5-cbc-pad, and rc5-cts algorithms. *The Internet Engineering Task Force Request For Comments*.

[40] Davies M http://www.wordfrequency.info.

[41] Fredman ML, Szemeredi E, Komlos J (1984). Storing a sparse table with o(1) worst case access time. *Journal of the ACM*.

[42] Kamara S, Papamanthou C, Roeder T (2011). Cs2: a searchable cryptographic cloud storage system.

